# Matrix Discriminant Analysis Evidenced Surface-Lithium as an Important Factor to Increase the Hydrolytic Saccharification of Sugarcane Bagasse

**DOI:** 10.3390/molecules24193614

**Published:** 2019-10-08

**Authors:** Ana Sílvia de Almeida Scarcella, Alexandre Favarin Somera, Christiane da Costa Carreira Nunes, Eleni Gomes, Ana Claudia Vici, Marcos Silveira Buckeridge, Maria de Lourdes Teixeira de Moraes Polizeli

**Affiliations:** 1 Departamento de Bioquímica e Imunologia, Faculdade de Medicina de Ribeirão Preto, Universidade de São Paulo. Bandeirantes Av., 3900, 14049-900 Ribeirão Preto, São Paulo, Brazil; asascarcella@yahoo.com.br; 2 Departamento de Biologia, Faculdade de Filosofia, Ciências e Letras de Ribeirão Preto, Universidade de São Paulo. Bandeirantes Av., 3900, 14040-901 Ribeirão Preto, São Paulo, Brazil; afsomera@gmail.com (A.F.S.); acvici@gmail.com (A.C.V.); 3 Departamento de Biologia, Instituto de Biociências Letras e Ciências Exatas, Universidade Estadual Paulista Júlio de Mesquita Filho. Cristovão Colombo Street, 2265, 15054000 São José do Rio Preto, São Paulo, Brazil; nuneschris@yahoo.com (C.d.C.C.N.); eleni@ibilce.unesp.br (E.G.); 4 Laboratório de Fisiologia Ecológica (LAFIECO), Departamento de Botânica, Instituto de Biociências, Universidade de São Paulo. Matão Street, 277, Cidade Universitária, 05508-090 São Paulo, Brazil; msbuckeridge@gmail.com

**Keywords:** lithium, sugarcane bagasse, saccharification, glycosyl-hydrolase, ToF-SIMS, surface ion distribution, second-generation ethanol, pretreatment

## Abstract

Statistical evidence pointing to the very soft change in the ionic composition on the surface of the sugar cane bagasse is crucial to improve yields of sugars by hydrolytic saccharification. Removal of Li^+^ by pretreatments exposing -OH sites was the most important factor related to the increase of saccharification yields using enzyme cocktails. Steam Explosion and Microwave:H_2_SO_4_ pretreatments produced unrelated structural changes, but similar ionic distribution patterns. Both increased the saccharification yield 1.74-fold. NaOH produced structural changes related to Steam Explosion, but released surface-bounded Li^+^ obtaining 2.04-fold more reducing sugars than the control. In turn, the higher amounts in relative concentration and periodic structures of Li^+^ on the surface observed in the control or after the pretreatment with Ethanol:DMSO:Ammonium Oxalate, blocked -OH and O^−^ available for ionic sputtering. These changes correlated to 1.90-fold decrease in saccharification yields. Li^+^ was an activator in solution, but its presence and distribution pattern on the substrate was prejudicial to the saccharification. Apparently, it acts as a phase-dependent modulator of enzyme activity. Therefore, no correlations were found between structural changes and the efficiency of the enzymatic cocktail used. However, there were correlations between the Li^+^ distribution patterns and the enzymatic activities that should to be shown.

## 1. Introduction

Demand for renewable fuels has considerably increased in recent years. Thus, there has been a significant increase of interest in sugarcane.

The most widespread crop in Brazil is sugarcane, with 391,767 thousand tons coming from the 2018/2019 harvest [1]. Sugarcane bagasse is composed of an elaborate arrangement of polysaccharides and proteins, combined with inorganic and organic ions acquired during different stages of culturing and processing [2]. Apart from these different sources of ions and the composition of the plant material, the pretreatment of sugarcane bagasse has become extremely important for determining the ionic composition of the substrate for the enzymatic saccharification. The cost and success of bioethanol production process from lignocellulosic biomass depends largely on the recalcitrant biomass itself, as well as on the repertoire of enzymes involved in the depolymerization of the constituent polysaccharides in the cell wall [3].

The pretreatment of sugarcane is crucial to ensure the conversion of polysaccharides into sugars for bioethanol production, since their chemical composition and physical structure are altered [4,5]. The different pretreatments can contribute to improving hydrolysis of the cell wall due to the rupture of the lignin structure and the connection with the rest of the biomass; removal of hemicellulose; and reduction of crystallinity and degree of polymerization of cellulose [6]. Several organic chemical changes have been researched using ToF-SIMS with great success [7]. However, pretreatments have the potential to alter the composition, relative concentration, and spatial distribution of alkaline and alkaline-earth metal ions on the substrate surface. This critical aspect possibly related to enzyme failure is still poorly researched. The mineral distribution on the surface of the substrate and the ionic properties of the surface area are key properties related to enzyme action because they are responsible for the surface charge, acidity, phase transfer, and the availability of binding and walking sites for enzymes [8,9]. Metals, alkaline metals, and alkaline earth metal ions are known to interfere positively or negatively in the enzymatic activity [8]. Therefore, it is necessary to map these changes and evaluate which ones interfere in enzyme function.

The importance of substrate as an ionic carrier is stressed in complex substrates such as sugarcane bagasse used in the second-generation ethanol industry.

The aim of this study was to analyze pretreatments of sugarcane bagasse to be used prior to enzymatic hydrolysis and to understand the anatomical factors related to our yield using ION-TOF. Furthermore, this work evaluated the neglected effect of current pretreatments on the composition, concentration and distribution of metallic ions on the surface of the sugarcane bagasse. Here, it was reported the extensive imaging analysis of different pretreatments of sugarcane bagasse used in second-generation ethanol production and their correlation with enzyme cocktail activity.

## 2. Results

### 2.1. Control-Milled Sugarcane Bagasse in Natura

Milled sugarcane bagasse *in natura* was chosen as control and subjected to direct saccharification. That material was also the initial material subjected to pretreatments to obtain a more suitable substrate for saccharification.

**Figure 1:**
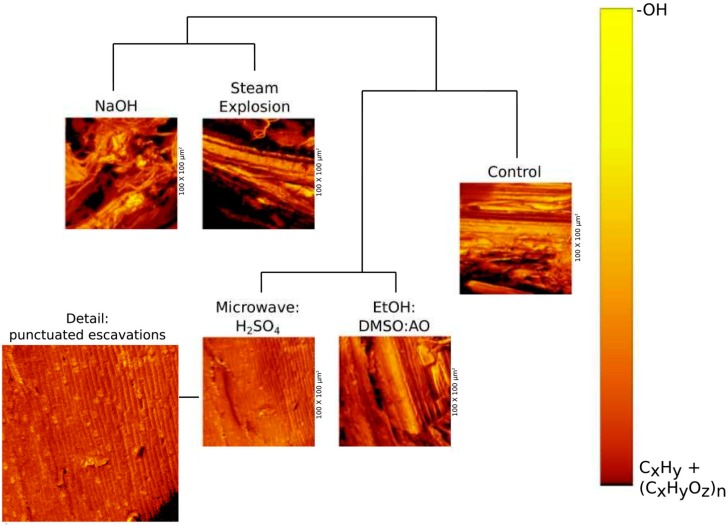
The overlapped signals of aromatics, (C_x_H_y_O_z_)_n_ residues and C_x_H_y_ chains generated from the ION-TOF analysis of sugarcane bagasse submitted to different pre-treatments. Control was material *in natura*. The dendogram was obtained comparing aromatics, (C_x_H_y_O_z_)_n_ residues and the chains of C_x_H_y_ distributions, total ion image surface entropy and roughness data of differently pretreated sugarcane bagasses at *p* = 0.05. NaOH and Steam Explosion pretreatments produced the most amorphous substrate because of the loss in periodicity of microfibril arrangements, while Microwave:H_2_SO_4_ essentially differed from Ethanol:Dimethyl Sulfoxide: Ammonium Oxalate (EtOH:DMSO:AO) pretreatment due to the production of slightly spherical excavations on the surface of the material, which can be observed in the chemical sputtering ion image. The images presented were obtained at negative mode and thus were dominated (88.3%) by overlapped signals of aromatics, (C_x_H_y_O_z_)_n_ residues and C_x_H_y_ chains. Yellow stains are superposed -OH and O^−^ chemical images.

**Figure 2:**
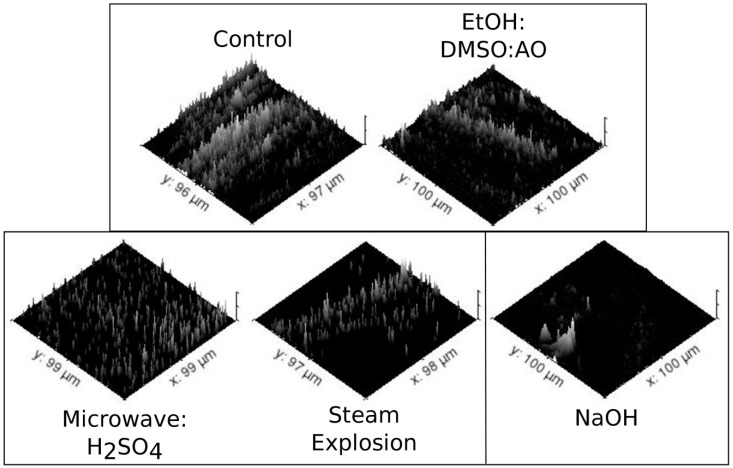
Mixture Discriminant Analysis and distribution patterns of Li^+^ on the surface of sugarcane bagasse. Pretreatments are grouped into conjuncts. The normalized spatial concentration patterns for released Li^+^, Li^+^[O^−^]_n_ and Li^+^[OH^−^]_n_ from the surface of pretreated sugarcane bagasse is presented. Parallel ridges of Li^+^ records were observed on the surfaces of control materials, Steam Explosion and Ethanol: Dimethyl Sulfoxide: Ammonium Oxalate (EtOH:DMSO:AO) pretreatments. The periodic arrangements in fringe and lattice structures positively correlated with the positioning of fibrils only for control and EtOH:DMSO:AO pretreatments, once Steam Explosion destroyed that arrangement.

Control presented a very compact and overlapped structure containing aromatics remnant of lignin, (C_x_H_y_O_z_)_n_ from carbohydrates and an almost uniform distribution of C_x_H_y_ from side groups. Sum images containing -OH and (C_x_H_y_O_z_)_n_ + C_x_H_y_ are shown in Figure 1. ToF-SIMS revealed a large number of impregnated ions in the structure including Na^+^, K^+^ and Mg^2+^, as well as Ca^2+^ covalently bounded (Ca^−^C_3_H_4_^+^) (Table 1). From those ions, Li^+^ covered 22.08% of -OH sites which were released together with Li^+^ sputtering (Table 2, Figure 2). Na^+^ and K^+^ spots were widespread on the surface (Table 1). Na^+^ presented clustered distribution forming small high-bulk density aggregates. Na^+^ was co-located with Cl^−^ and PO^−^, PO_2_^−^ and PO_3_^−^ sites. However, only 48.7% of PO^−^, PO_2_^−^ and PO_3_^−^ sites, co-located with Na^+^, were sputtered from the surface of the sugarcane bagasse. All other elements had a random distribution. The density of H^+^ on the surface was very large. The negative mode sputter eroded ions at a mass range of 160–180 Da related to surface glucose, arabinose, and xylose units of the exposed cellulose and hemicellulose, respectively.

### 2.2. Steam Explosion Pretreatment

At positive mode, the mass spectrometric analysis of sugarcane bagasse subjected to steam explosion evidenced a decrease in the concentrations of metal and semi-metal ions (Table 1). The records for O^−^, Cl^−^ and -OH were enriched 3.1-fold. The resulting chemical image of the surface of the substrate evidenced the increase in the exposure of compounds at 279–280 Da (benzoate) and at 417 Da (syringaresinol) from lignin. The pretreatment also increased 2.47-fold the (C_x_H_y_O_z_)_n_ at 160–180 Da (glucose and xylose from cellulose and hemicelluloses, respectively). The steam explosion produced 2.80-fold higher dimethyl dialkyl ammonium (DDA) amounts at random distribution than other pretreatments. In turn, Li^+^ was dramatically reduced (2.20-fold) compared to control and presented random distribution (Figure 2). At negative mode, the exposure of anions and sites for an enzymatic attack such as -OH, O^−^, (C_x_H_y_O_z_)_n_ and aromatics showed a 2.31-fold mean increase in signal intensity (Figure 1). Microscopic changes in fiber were evident by chemical image, presenting lattice deformation even at micrometer scale but without complete fibrils dismemberment and their periodic fringe ordering, whose remnants occupied 47% surface area.

### 2.3. Microwave:H_2_SO_4_ Pretreatment

Microwave:H_2_SO_4_ pretreatment decreased metal ions on the surface of the substrate (Table 1). At negative mode, the chemical image presented aromatics related to lignin superposed with C_x_H_y_ signals. The image showed 1.22-fold lower records for (C_x_H_y_O_z_)_n_ on the surface than control, revealing higher hindering of celluloses and hemicelluloses. It is worth mentioning the low abundance of ions randomly distributed on the surface, 11.8% lower than control, but 43.2% higher than Steam Explosion. The surface of that pretreated substrate had 2.60-fold less DDA than the Steam Explosion and 28.8% less Li^+^ than the control. The periodic structure of fibrils was 97.2% preserved rather than prior to treatment. Microwave:H_2_SO_4_ produced a singular effect: the substrate pretreated surface showed 72 roughly circular excavations for 100 × 100 µm^2^ area. Excavations presented an average radius of 0.72 µm and 2.45 nm depth, which were extremely rich in -OH and O^−^ (Figure 1). These excavations presented 68.4% of its surface covered by -OH and O^−^from cellulose and hemicelluloses, and were devoid of Li^+^, Ca-C_3_H_4_^+^, Mg^2+^, and -NH_3_^+^.

### 2.4. Ethanol:Dimethyl Sulfoxide:Ammonium Oxalate (EtOH:DMSO:AO) Pretreatment

The chemical image confirms the increase in the exposure of aromatic compounds and 1.21-fold increment in the exposal of (C_x_H_y_O_z_)_n_ at positive mode, followed by a 1.09-fold rise in the amounts of -NH_3_^+^, -CN and -CNH groups overlapped with (C_x_H_y_O_z_)_n_ on the substrate surface. These results pointed to overlaps between proteins and carbohydrates at the substrate surface. The pretreatment randomly added DDA and produced a low ionic average count (Table 1). At negative mode, the (C_x_H_y_O_z_)_n_ and aromatic exposure showed 1.30-fold higher signal intensity than the control because of the better exposure of fibrils’ after that pretreatment (Figure 1).

### 2.5. NaOH Pretreatment

The chemical image revealed the complete deformation of the lattice structures observed in the control (Figure 1). Only 8.7% of the remnant parallel arrangements and lattice structures at the substrate surface were preserved in a 100 × 100 µm^2^ area. That pretreatment also produced the biggest enrichment in Na^+^ and F^−^ recorded, equivalent to a 1.73-fold mean increase relative to the control (Table 1). The increase in F^−^ and K^+^ was routed to impurities in the NaOH solution (data not shown), while Na^+^ enrichment can be a possible side effect of the pretreatment. In addition to the increased exposure of (C_x_H_y_O_z_)_n_ and C_x_H_y_, aromatics at 279—280 Da identified as benzoate and bi-phenolic residues from lignin. Li^+^ was only observed in aggregates with DDA (Figure 2), but not on the remnant surface. Despite the higher relative concentrations of Na^+^ and K^+^, all other ion concentrations reduced (Table 1). Ions were distributed in lower numbers of aggregates occupying a reduced surface of the substrate. Li^+^ presented a decrease of 1.41-fold in relation to the control (Table 1, Figure 2). That was observed with Microwave:H_2_SO_4_, but it was lower than those obtained with Steam Explosion and EtOH:DMSO:AO. Compared to the control, -OH and O^−^, increased 1.65-fold (Figure 1). That was the biggest gain achieved with -OH exposition. Only the circular excavations verified in Microwave:H_2_SO_4_ pretreatment presented higher amounts of -OH exposed. However, circular excavations verified after Microwave:H_2_SO_4_ pretreatment occupied only 68.4% a 100 × 100 µm^2^ area, while the area occupied by -OH after NaOH pretreatment represented 97.4% of the surface analyzed.

### 2.6. Clustering Pretreatments Using Discriminant Analysis with Machine Learning—Anatomical Parameters

Figure 1 shows the comparison of surface anatomical parameters (*p* = 0.05) of sugarcane bagasse differently pretreated. Aiming to compare all pretreatments, matrix discriminant analysis with machine learning was used, due to the non-linearity of data and the very complex nested matrix of parameters obtained. The surface structure properties analyzed were the percentage of the area occupied by C_x_H_y_, (C_x_H_y_O_z_)_n_ and aromatics (i.e., sugars and lignin) surface area covered by radicals (-OH and O^−^), which are related to hydroxyl groups used by glycosyl-hydrolases during their action; the surface area occupied by periodic, parallel, and lattice structures containing C_x_H_y_ and (C_x_H_y_O_z_)_n_; and surface entropy, waviness, and roughness. These parameters were automatically collected using the free and open-source softwares ImageJ and Gwyddion.

According to these parameters, NaOH and Steam Explosion pretreatments clustered together and produced the most amorphous substrate. It happened due to the loss of periodicity of the carbohydrate surface arrangements. On the other hand, Microwave:H_2_SO_4_ differed from the pretreatment with EtOH:DMSO:AO, because of the production of slightly spherical excavations on the surface of the material. The fibrils and microfibrils, observed in the control, presented intense cover-up by organic matter, visible in the images as amorphous deposits containing -OH and (C_x_H_y_O_z_)_n_ residues and chains of C_x_H_y_ (Figure 1). The estimated differential entropy for the control surface was equal to −20.46 nats (natural units of information) with a deficit of 0.16266 nats, because of those roughly amorphous deposits.

### 2.7. Clustering Pretreatments Using Discriminant Analysis with Machine Learning – Ionic Parameters

The matrix discriminant analysis of the ionic parameters from the surface of the substrates clustered the following samples: EtOH:DMSO:AO pretreatment and Control; Steam Explosion and Microwave:H_2_SO_4_; and finally, isolated the NaOH pretreatment in a single group (Figure 2, Table 1). Using a jackknife procedure, deleting single ionic parameters before running the matrix discriminant analysis with machine learning, the ionic parameters related to Li^+^ (Table 2) were identified as responsible by 37,6% of these results. Figure 2 showed the results of the matrix discriminant analysis with computerized images of Li^+^ obtained using the Gwyddion software.

Steam Explosion and Microwave:H_2_SO_4_ pretreatments produced lower counting of aggregates, the lowest total area occupied by Li^+^ and the lowest average size for Li^+^ aggregates. However, both differed in DDA, only present after the Steam Explosion pretreatment (Table 1), and -OH-enriched excavations presented only after Microwave:H_2_SO_4_ pretreatment (Figure 1).

The NaOH pretreatment produced higher counts, percentage area and average size of aggregates of Li^+^, but all Li^+^ observed was not distributed accompanying fibrils of carbohydrates such as in Steam Explosion or control. In turn Li^+^ presented random distribution and a very low abundance on the surface of the substrate, but one high bulk density aggregate of 13.79 µm^2^ in a 100 × 100 µm^2^ chemical image area.

### 2.8. Performance of the Enzyme Cocktail Versus Pretreatment-the Enzymatic View Versus the Matrix Discriminant Analysis of Anatomical and Ionic Composition on the Surface of the Substrate

**Figure 3:**
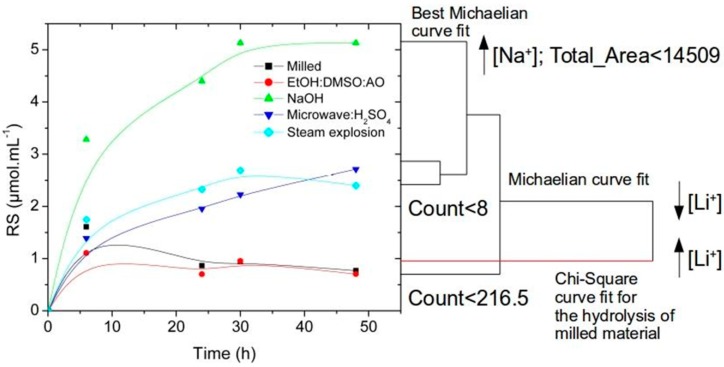
Time-dependent release of reducing sugars during the enzymatic hydrolysis of sugarcane bagasse. Generalized additive model (GAM) curves for reducing sugars (RS) released and the similarity analysis among enzyme cocktail activities were shown with the major traits related to the structural differences among each pretreatment using Mixture Discriminant Analysis (MDA) at *p* = 0.05.

The best saccharification yield was obtained after NaOH pretreatment, which increased the release of reducing sugars by enzymatic saccharification in 2.04-fold more than the control at 10 h intervals (Figure 3). The right side of Figure 3 shows a tree made using the results from the MDA analysis of treatments used in each saccharification assay. The tree was placed next to the results of the saccharification assays to highlight the similarities between enzyme responses and MDA analysis results. Analysis was done at *p* ≤ 0.05. It was noted the relevant events in the dendrogram, such as the non-Michaelian branch related to milled material, the counts of lithium-ion clusters on the surface of the materials, the qualitative richness of Sodium observed in NaOH treatment, the total area free from metal ions less than 14,509 µm^2^, and the qualitative changes in lithium-ion concentration on the surfaces analyzed. These raw data are in Table 1. Steam Explosion and Microwave:H_2_SO_4_ clustered in an intermediate subset during enzymatic hydrolysis increasing 1.74-fold the reducing sugars release at 10 h intervals compared to control. In turn, a 1.1-fold decrease in the production rates of reducing sugars was identified after EtOH:DMSO:AO pretreatment when compared to control. EtOH:DMSO:AO and control also presented time-dependent decay for the release of reducing sugars after 10 h intervals.

### 2.9. Matrix Discriminant Analysis (MDA) of Anatomical and Ionic Parameters from the Surface of the Substrate Versus the Enzymatic View—Correlation Between Discriminant Analysis and Saccharification Yields

MDA for the anatomical parameters, including entropy, roughness, exposition of aromatics from lignin, (C_x_H_y_O_z_)_n_ residues and C_x_H_y_ chains from carbohydrates, and microfibril and fibril arrangements (Figure 1), did not correlate with data obtained in this study about saccharification yields at *p* = 0.05. As enzymes are good topological recognizers, the information did not correlate with the topological view of the enzymes. These results clustered pretreatments as the least-squares method for the curves of time-dependent release of reducing sugars at *p* = 0.05 (Figure 3).

### 2.10. Enzyme Cocktail Response to Metal Ion Salts in Solution

As ionic parameters produced the same cluster profile for pretreatments as the saccharification and time-dependent releases of reducing sugars, the effects of the ions observed on the surface of the substrate were evaluated to check their impact on the saccharification when in solution. All ions tested activated glycosyl-hydrolases when in solution (Table 3), but decreased laccase activity up to 87% (for NH_4_F). Therefore, it can be inferred that these ions negatively interfere in lignin degradation, but acted as non-essential activators for hemicellulases and cellulases when in solution. These results are opposed to the correlation observed between surface-bounded ions and saccharification.

## 3. Discussion

Glycosyl-hydrolases need to deal with a very complex surface area rich in C_x_H_y_ and -OH sites for reaction. In addition to the ratio between hydrophobic areas and reaction sites containing -OH and O^−^, the presence of several bounded metal ions on the surface of the substrate resulted in a new level of complexity to be considered for the development of a pipeline linking pretreatment and cocktail.

To be analyzed by ToF-SIMS, the material was deposited two-dimensionally on the disc for ion excavation. The specific surface area did not show visible relations with the enzyme hydrolysis. So, new aspects were evaluated for these materials. Interpreting the surface area is difficult, as some ions occupied the entire surface area evaluated for each material, such as Calcium and Chlorine ions, which can be seen in Table 1. Metal cation-free areas were rare, such as the 14,509 um^2^ observed in NaOH treatment. No material had Anion-free areas at that micrometric scale. In addition, each material has its own surface area. The overlapped surface areas occupied by each metal ion and hydroxyl sites are more valuable, and the constant overlap between hydroxyl radicals and lithium ions were observed as exclusive for these materials. Perhaps the excess of other ions made it impossible to see overlaps among other cations and the hydroxyl-rich regions, spreading to areas containing C-H-rich hydrophobic regions. For example, the amounts of potassium, sodium, and the anions fluorine and chlorine were so high that they occupied the entire surface evaluated.

Contrary to expectations, the increased exposure of fibrils, Steam Explosion, and NaOH pretreatments did not answer equally during the enzymatic hydrolysis. On the other hand, Steam Explosion presented a saccharification statistically equivalent to that observed after Microwave:H_2_SO_4_ pretreatment. This pretreatment did not produce major changes in the arrangement of the microfibrils such as Steam Explosion, neither similar information about surface-sugars were observed, as the ones described above. However, both decreased the abundance and area occupied by metal-ions on the surface of the substrates compared to control. In particular, the areas containing exposed -OH and O^−^ free of Li^+^, proved a recurring property in both. This property was shared by the substrates subjected to NaOH treatment.

The NaOH pretreatment increased surface-Na^+^ while reducing surface-Li^+^, reducing the surface acidity while increasing O^−^ and -OH on the surface area. The combination of amorphous structures, loss of periodicity in the spatial distribution of carbohydrates, and the reduced surface-bounded Li^+^ together with the very large amounts of O^−^ and -OH sites were possibly good factors to develop productive E S complexes [10], because the strong increase in saccharification compared to other pretreatments and control.

The overall ionic cleaning of the surface increased the exposure of -OH and O^−^, but the ion that most influenced the pattern of discrimination among substrates was lithium, which was sputter eroded with -OH, implying covering of OH by that ion. Although the changes in the concentration and distribution of nonessential ion activators were not limited to Li^+^, the best results during the degradation of the substrate were obtained just after pretreatments capable of removing Li^+^ from the -OH and O^−^ surfaces of the substrate, i.e., NaOH, Steam Explosion and Microwave:H_2_SO_4_ pretreatments. These pretreatments increased 1.23-fold mean the available Li^+^-free -OH and O^−^ for ion sputtering than the control material.

Using only the main discriminating factors related to the ionic composition and distribution, i.e., the counting of Li^+^ aggregates, the distance between Li^+^ spots and the average size of Li^+^ clusters, it was possible to identify relationships between Li^+^ distribution and enzyme activity.

Steam Explosion pretreatment produced an almost uniform pattern of Li^+^ distribution accompanying fibrils, while Microwave:H_2_SO_4_ produced random distribution patterns. Therefore, different distribution patterns, with statistically similar counts of aggregate and distancing of Li^+^ on the surface of pretreated sugarcane bagasse, resulted in statistically similar production rates of reducing sugars by the presented enzyme cocktail. Once Steam Explosion and Microwave:H_2_SO_4_ do not have structural relationships related to fibril arrangement, the only aspect which linked both pretreatments, were the surface concentration and distribution of Li^+^.

In turn, the pretreatment with NaOH produced the highest aggregation of Li^+^, but the lowest concentration and the largest distribution of Li^+^ on the substrate surface. It allowed the exposure of –OH, possibly, due to the reduced formation of coordination groups between Li^+^ and -OH, which results in the highest sputtering of Li^+^ free of OH and O^−^ radicals from the cane surface. NaOH also reduced the periodic arrangement of fibrils, -OH^−^ and O^−^ enriched sites and reduced the surface occupied by Li^+^, which positively correlated with the highest activity observed on pretreated substrates. Once Li^+^ on the surface of the substrate can block -OH as revealed by the sputter erosion of Li^+^ together with -OH, surface Li^+^ can negatively interfere in the enzyme performance because glycosyl-hydrolases require free -OH to react [11]. This hypothesis could be risen using the results about the correlation between the increases of surface-bounded Li^+^ and covering of -OH sites with the decrease in enzyme activity. Given the differences between the effects of ions in solution and the results from the matrix discriminant analysis, ions adhered to the substrate could affect the enzyme action differently from those in suspension. Thus, it could be hypothesized that the real substrate for the enzyme is an ionic polysaccharide coordination system.

An important feature found during the enzymatic hydrolysis of control materials was the great performance of the cocktail response during the first 10 h of the assay. Enzyme performance was second only to that shown after NaOH pretreatment of the substrate. Apparently, a depolymerase adaptation to control materials, i.e., the original substrate found in nature, allowed the rapid recognition of binding sites for a rapid phase transfer, resulting in large amounts of productive interactions, which did not exceed only those observed for the assays using NaOH-pretreated substrate. Therefore, the majority of pretreatments are only important during prolonged hydrolysis times.

On the other hand, the EtOH:DMSO:AO pretreatment produced an ionic surface very similar to that of control according to matrix discriminant analysis, especially regarding the pattern of surface distribution of Li^+^. Nevertheless, the first 10 h of enzymatic attack recorded for this pretreatment was the least productive. As DDA was a substantial change in that substrate, it could be inferred a failure in the substrate recognition by enzymes. Although the solubility of the substrate could be improved after that pretreatment, caution is needed when adding methyl and amino groups in the carbohydrate structure, since its recognition as a substrate for enzymatic cocktails can be a problem.

EtOH:DMSO: AO pretreatment and control presented a strong decrease in reducing sugars after 10 h reaction times even without microbial activity. It was difficult to determine the cause for those decays. Once both control and EtOH:DMSO:AO pretreated biomass clustered together in every matrix discriminant analysis of chemical images, the observed decay can be related to ions trapped on the substrate surface and released into the reaction solution inducing reverse reactions or reducing sugar precipitation. The causes for those phenomena remain hindered by a very complex mixture produced during the saccharification.

It can be assumed that there is a complex causal mechanism controlled by phase-transfer of enzymes and ions affecting cooperative, competitive and deinhibitory processes, anchimeric assistance and [ion]^−^ dependent induced-fit behavior of glycosyl hydrolases. Thus, it could be concluded that lithium was an activator in solution, but its pattern of presence and distribution in the substrate can act as an inhibitor. These results pointed to a phase-dependent action for alkali metal ions in the enzymatic activity.

## 4. Materials and Methods 

### 4.1. Control—Milled Sugarcane Bagasse in Natura

Sugarcane bagasse was provided by Sugar and Alcohol Mills (Ribeirão Preto, São Paulo, Brazil). It was washed in tap water to remove reducing sugars, dried at 50 °C, and then milled in a knife mill SL 32 (SOLAB), 25 mesh. The material obtained was used as the control for the described pretreatments.

### 4.2. Sugarcane Bagasse Pretreatments

Sugarcane bagasse was submitted to four different pretreatment types. For all the pretreated sugarcane bagasse the resulting material was washed with deionized water until the complete removal of reducing sugars. The reducing sugars released were monitored using the 3,5-Dinitrosalicylic Acid (DNS) method [12]. After washing, the material was dried at 50 °C and stored at room temperature.

#### 4.2.1. Steam Explosion Pretreatment

Milled sugarcane bagasse *in natura* was maintained in steam water at 14 kg.cm^−2^ for 8 min, followed by rapid steam water expansion.

#### 4.2.2. Microwave:H_2_SO_4_ Pretreatment

Microwave pretreatment was made according to Moretti et al. [13] with minor changes. A sample of 10 g of sugarcane bagasse was immersed for 24 h in, a solution of 0.05 M H_2_SO_4_ and glycerol. After that, this sample was transferred to a 250-mL round-bottom flask into a microwave oven which was connected to a spinning reflux condenser. The released sample was irradiated at 2450 MHz for 5 min. An infrared thermometer was used to detect the temperature. Further, 30 mL of distilled water was added to the material, mixed, filtered and this suspension was used to determine the amount of reducing sugars. 

#### 4.2.3. Ethanol: Dimethyl Sulfoxide: Ammonium Oxalate Pretreatment

Cell wall components from sugarcane bagasse *in natura* were fractionated using the protocol described by Lima et al. [14]. For the removal of soluble sugars, samples of sugarcane bagasse (1 g) were incubated in 20 mL 80% ethanol at 80 °C for 20 min under constant stirring. The resulting material was centrifuged (11,000× *g*) for 15 min, and the supernatant was discarded. This step was repeated six times, and the resulting precipitate was washed with 20 mL of distilled water and dried overnight in an oven at 50 °C. Starch was removed incubating the dried material in 20 mL 90% Dimethyl Sulfoxide (DMSO) at 90 °C for 24 h, while pectins were extracted incubating the starch-free material in 20 mL ammonium oxalate solution, pH 7.0 at 80 °C for 3 h.

#### 4.2.4. NaOH Pretreatment

50% dry mass of EtOH:DMSO:AO treated materials were hydrolyzed using NaOH to remove hemicelluloses [14]. The material was hydrolyzed at room temperature using a sequence of three steps: (1) 1-h hydrolysis time using 20 mL 0.1 M NaOH: 0.1 M sodium borohydride; (2) 1-h hydrolysis time at room temperature using 20 mL 1.0 M NaOH: 0.1 M sodium borohydride; and (3) 1-h hydrolysis time at room temperature using 20 mL 4.0 M NaOH: 0.1 M sodium borohydride.

### 4.3. Enzymatic Hydrolysis

The enzyme cocktail applied to achieve cell wall degradation used 0.122 U laccase (*Trametes versicolor*); 7 U xylanase (*Malbranchea pulchella* expressed in *Aspergillus nidulans*) [15]; 5 U endoglucanase (*Aspergillus terreus* expressed in *A. nidulans*) [16], 14 U cellobiohydrolase (*Aspergillus niveus* expressed in *A. nidulans*) [17], and 9 U β-glucosidase (*Aspergillus niger*) per gram of lignocellulosic biomass. Materials were suspended into 7 mL of 50 mM sodium citrate buffer, pH 5.0. Hydrolysis was conducted at 55 °C and 110 rpm during 48 h. Reducing sugars were determined using DNS method [12].

### 4.4. Effects of Dissolved Salts on Specific Enzyme Activities

The effects of the salts observed on the surface of sugarcane bagasse upon the enzyme cocktail activities were analyzed in 10 mM final concentration: NH_4_F, NaH_2_PO_4_, MgCl_2_.6H_2_O, NH_4_Cl, CaCl_2_, KCl, LiCl, Na_2_SO_4_, MnCl_2_·4H_2_O, NaCl, KH_2_PO_4_, BaCl, and Zn(NO_3_)_2_. Endoglucanase and xylanase were measured using the substrates β-glucan and xylan beechwood, respectively. Determination of reducing sugars released used the DNS method [12] and glucose and xylose as controls for activity determination of endoglucanase and xylanase respectively. Cellobiohydrolases and β-d-glucosidases were determined by the cleavage of ρ-nitrophenyl-cellobioside (ρNPC) and ρ-nitrophenyl-β-d-glucopyranoside (ρNPG), respectively. ρ-Nitrophenol was used as standard. Laccase activity was determined using syringaldazine as substrate [18]. The oxidation of syringaldazine to quinone at room temperature was measured by the increase in the absorbance at 525 nm during 5-min of reaction. All assays used 50 mM sodium citrate buffer pH 5.0 at 55 °C. One unit of enzymatic activity was defined as the amount of enzyme that released 1 μmol min^−1^ of products. All experiments were performed in triplicate.

### 4.5. Chemical Image Analysis

Sugarcane bagasse was surfaced-analyzed using an ION-TOF TOF.SIMS 5 instrument at ION-TOF-TasconGmbh (Heisenbergstr, Münster, Germany) using Bi_3_^+^ as primary ion for the analysis of organic and inorganic materials. The primary ion energy was 30 keV, analysis current of 0.8 pA, analysis area from 25 × 25 μm^2^ to 100 × 100 μm^2^ and measurement time of 100 s. The measurement conditions used positive mode, suitable for metal ions and non-metallic salts and compounds containing amino groups, and the negative mode, used for ionization of carbohydrates (loss 1 H^+^ or more protons). ION-TOF TOF.SIMS 5 instrument (ION-TOF GmbH, Münster, Germany) was controlled by the SurfaceLab software suite. This software was used for data acquisition and analysis using the included spectrum library for sample identification.

After a sum of the three-color channel images into a single channel, TIFF image files were converted to the standard 8-bit gray-scale file format using the ImageJ software [19]. Before analysis, each image for each ion and anatomical structure were standardized using the threshold method included in the ImageJ software. Standardization employed the IJ_IsoData algorithm and produced two-dimensional maps in red and black colors.

ImageJ software was used to count both, ionic spots numbers and intensities using densitometric analysis. The area recovered with ions, the diameter of ionic aggregates and the color intensities of ionic aggregates and areas covered with ions were also measured using the standardized images and the ImageJ software.

For anatomical analysis, the Gwyddion software [20] was used to measure the follow parameters: texture, roughness and waviness, the diameter of fibrils and fibril aggregates in lattice structures, and the diameter of excavations caused by microwave:acid treatments.

All anatomical and ionic data were summarized in matrices and analyzed using discriminant analysis.

### 4.6. Statistical Analysis

Data automatically collected using ImageJ and Gwyddion software were used to generate a numerical matrix for each treatment. Matrices summarizing ionic parameters at the surface of substrates and anatomical data included the ionic composition measured using ToF-SIMS and the relative concentration of each ion, ionic aggregate counts, total surface area occupied by ionic aggregates, the average area of ionic aggregates for each target ion, and the values measured for texture, roughness and waviness, the diameter of fibrils and fibril arrangements in lattice structures, and the diameter of punctuated excavations. Discriminant analysis with machine learning using R 3.3.1 [21] was employed to compare each matrix summarizing the ionic and anatomical parameters produced by each treatment. The discriminating analysis used the Mixture Discriminant Analysis method from the MDA [22] package at *p* = 0.05. This method is suitable to deal with difficult data sets. The discriminant analysis of the parameters in each pretreatment was made using function training, considering the levels = pretreatments. MDA greatly succeeded during the discrimination of all pretreatments because the non-normal distribution of data. The analysis produced a string of pretreatment names s when neighbor pretreatments were more similar than distant ones. A matrix was produced using the strings by assigning 1 to each neighbor pretreatment name in the resulting string and 0 to no neighbor. These binary matrices were used to generate a tree clustering pretreatment using R 3.3.1 and the tree package [21]. In order to estimate the best parameter to discriminate each pretreatment, data were jackknifed, which allowed the leaving of one column out at a time, and the discriminant analysis of the matrix was re-run. The parameter used to discriminate the major percentage of pretreatments in a string produced by matrix discriminant analysis was considered the best discrimination parameter to be used in the process.

Comparison among curves of enzymatic saccharification used the least-squares method implemented in R 3.3.1 [21]. All comparisons among time-dependent enzymatic saccharification were done at *p* = 0.05.

## 5. Conclusions

Anatomical parameters were not related to saccharification yields of sugarcane bagasse. However, a strong factor affecting the performance of the enzyme cocktail was lithium coordinated with the substrate. So, it can be concluded that Lithium was an activator in solution, but its presence and distribution pattern on the substrate can act as an inhibitor. These results pointed to a phase-dependent action for alkaline metal ions on enzyme activity.

Control and substrates produced by EtOH:DMSO:AO pretreatment also presented a time-dependent decay of reducing sugars in the solution possibly related to ion-dependent precipitation or reverse reactions. The cause for that phenomenon remains hindered by a very complex mixture produced during the saccharification process. Once all materials analyzed presented very similar ionic composition, but differed in relative concentration and distribution of lithium, it was necessary to focus the analysis and absolute quantification of ions and ionic rates related to these enzyme responses. It can be surmised that there is a complex causal mechanism controlled by phase-transfer of enzymes and ions affecting cooperative, competitive and deinhibitory processes, anchimeric assistance, and [ion]-dependent induced-fit behavior of glycosyl-hydrolases. So, it is necessary to develop a new integrative kinetics approach to deal with all these aspects related to ion-polysaccharide hydrolysis.

It is likely that these pretreatments will not generate such predicted structural responses as revealed by specific surface area data (Control: 5751.60 µm^2^/ng; Steam explosion: 4375.16 µm^2^/ng; Microwave:H_2_SO_4_: 6544.19 µm^2^/ng; EtOH:DMSO:AO: 5235.62 µm^2^/ng; NaOH: 5347.32 µm^2^/ng). This problem prevented any expected correlation between pretreatment and saccharification response, but opened new possibilities for screening new characteristics. In this case, statistical indications of the importance of lithium distribution on enzymatic activity during the screening for correlations were found. It is not aware of investigations into the influence of cations adsorbed on the substrate. It only knows the effect of cations in solution. It is expected that these novelties open previously unexplored paths for the study of the influence of complexed or adsorbed cations on enzymatic activity assays, contributing to the development of more efficient enzymatic cocktails.

## Figures and Tables

**Table 1:** Ion particle analysis after automated color threshold.

Ion/ Compost	Control *in Natura*			Steam Explosion Pretreatment			Microwave:H_2_SO_4_ Pretreatment			EtOH:DMSO:AO ^a^ Pretreatment			NaOH Pretreatment		
	Count (N)	Area (μm^2^)	Average Size (μm^2^)	Count (N)	Area (μm^2^)	Average Size (μm^2^)	Count (N)	Area (μm^2^)	Average Size (μm^2^)	Count (N)	Area (μm^2^)	Average Size (μm^2^)	Count (N)	Area (μm^2^)	Average Size (μm^2^)
Li^+^	353	53.857	0.150	88	0.054	0.027	63	0.027	0.027	320	30.879	0.097	16	4.569	0.286
Na^+^	71	349.574	6.221	35	185.866	5.310	121	376.032	3.108	61	383.557	6.288	23	374.151	16.267
K^+^	19	432.026	26.729	50	254.450	5.089	94	364.368	3.876	38	387.346	10.193	15	376.596	25.106
Mg^2+^	144	249.479	2.058	107	116.690	1.091	777	124.457	0.160	570	105.242	0.185	119	178.072	1.496
Ca-C_3_H^4+^	44	407.019	11.957	122	231.607	1.898	28	487.885	17.424	83	393.608	4.742	42	316.961	7.547
F^−^	197	278.208	4.107	209	167.511	0.801	778	168.263	0.216	299	244.991	0.819	46	386.540	8.403
Cl^−^	62	369.058	7.128	135	164.850	1.221	65	457.812	7.043	224	234.241	1.046	53	367.540	6.935
DDA_b_	0	0.000	0.000	9	0.403	0.045	7	0.215	0.031	7	0.403	0.058	9	2.365	0.263

^a^ Ethanol:Dimethyl Sulfoxide:Ammonium Oxalate. ^b^ Dimethyl Dialkyl Ammonium.

**Table 2:** Percentage of -OH sites uncovered by Li^+.^

Pretreatment	Li^+^-free-OH Area (%) *
Control (*in natura*)	77.92
EtOH:DMSO:AO ^a^	78.50
Steam Explosion	84.98
Microwave:H_2_SO_4_	85.00
NaOH	84.16

^a^ Ethanol:Dimethyl Sulfoxide:Ammonium Oxalate. * The percentage of lithium-free surface area observed after NaOH pretreatment was due to the presence of a heavy Li^+^ aggregate located in only one site on the surface of the substrate. The Li^+^ distribution for Steam Explosion and Microwave:H_2_SO_4_ were widespread on the surface of the substrate generated after both pretreatments (Figure 2).

**Table 3:** Effects of ion salt in solution on the enzyme activities.

Ion Salts	Laccase	Xylanase	Endoglucanase	Cellobiohydrolase	β-Glucosidase
	(%)	(%)	(%)	(%)	(%)
NH_4F_	12.34	141.68	109.84	126.50	135.70
NaH_2_PO_4_	37.56	146.81	99.06	100.10	141.80
MgCl_2_∙6H_2_O	41.36	137.39	61.40	110.90	144.79
NH_4_Cl	43.39	155.04	23.44	103.80	138.54
CaCl_2_	43.07	147.14	120.15	119.30	144.23
KCl	42.72	148.24	140.31	109.50	146.57
LiCl	42.24	130.59	133.59	101.60	148.55
Na_2_SO_4_	29.41	181.60	110.78	137.30	152.23
MnCl_2_∙4H_2_O	30.01	216.97	164.68	129.00	143.83
NaCl	38.20	175.46	134.68	129.70	150.57
KH_2_PO_4_	33.56	162.18	124.22	148.30	143.17
BaCl	34.04	139.41	123.43	144.30	143.57
Zn(NO_3_)_2_	40.48	122.86	119.84	140.10	137.07

Control (without ions) corresponded to 100%.
